# Caterpillar-Inspired Multi-Gait Generation Method for Series-Parallel Hybrid Segmented Robot

**DOI:** 10.3390/biomimetics9120754

**Published:** 2024-12-11

**Authors:** Mingyuan Dou, Ning He, Jianhua Yang, Lile He, Jiaxuan Chen, Yaojiumin Zhang

**Affiliations:** 1 College of Mechanical and Electrical Engineering, Xi’an University of Architecture and Technology, Xi’an 710055, China; doumingyuann@xauat.edu.cn (M.D.); hllnh2013@163.com (L.H.); chenpalmer@xauat.edu.cn (J.C.); zhangyaojiumin@foxmail.com (Y.Z.); 2 College of Automation, Northwestern Polytechnical University, Xi’an 710072, China; yangjianhua@nwpu.edu.cn

**Keywords:** bio-inspired locomotion, worm robot, gait generation

## Abstract

The body structures and motion stability of worm-like and snake-like robots have garnered significant research interest. Recently, innovative serial–parallel hybrid segmented robots have emerged as a fundamental platform for a wide range of motion modes. To address the hyper-redundancy characteristics of these hybrid structures, we propose a novel caterpillar-inspired Stable Segment Update (SSU) gait generation approach, establishing a unified framework for multi-segment robot gait generation. Drawing inspiration from the locomotion of natural caterpillars, the segments are modeled as rigid bodies with six degrees of freedom (DOF). The SSU gait generation method is specifically designed to parameterize caterpillar-like gaits. An inverse kinematics solution is derived by analyzing the forward kinematics and identifying the minimum lifting segment, framing the problem as a single-segment end-effector tracking task. Three distinct parameter sets are introduced within the SSU method to account for the stability of robot motion. These parameters, represented as discrete hump waves, are intended to improve motion efficiency during locomotion. Furthermore, the trajectories for each swinging segment are determined through kinematic analysis. Experimental results validate the effectiveness of the proposed SSU multi-gait generation method, demonstrating the successful traversal of gaps and rough terrain.

## 1. Introduction

Tasks such as pipeline inspections and earthquake rescues often require navigating narrow and unstructured terrains. Segmented robots excel in these environments due to their compact sizes and unique modes of movement. In contrast, other types of robots are generally less efficient [1] and face challenges in achieving a compact design. Recent research has introduced novel segmented robot structures that incorporate both serial and parallel connections. However, significant potential remains to further enhance the performance of these mechanisms and improve their adaptability in unstructured environments.

Due to their large contact area and low center of gravity, limbless animals maintain continuous contact with the substrate [2], resulting in high stability during movement. Consequently, many researchers have designed segmented robots to mimic these animals, aiming to replicate their locomotion performance and terrain adaptability, as seen in earthworms [3], inchworms [4], and snakes [5]. Various studies have applied the serpentine gait of snakes [6,7] and the rectilinear gait, which is similar to the earthworm’s gait, to segmented robots [8,9,10]. The serpentine gait, however, requires more space for movement [11]. During rectilinear and earthworm-like movements, all segments remain in contact with the ground [12]. Although the speed achieved in Hopkins’ work [10] was quite high, reaching 167 mm/s in a high-traction forward gait, this gait exhibits lower adaptability to unstructured terrain.

Unlike other limbless animals, only a few segments of a caterpillar are involved in the swinging phase [13,14], while most segments remain anchored to the substrate during the stance phase [15,16]. Furthermore, the swinging segments are lifted away from the substrate, reducing friction and enabling caterpillars to climb more stably. This type of movement, characterized by minimal space requirements and high stability, suggests better adaptability to various environments and has become an important reference for the motion design of segmented robots.

Segmented robots are generally classified based on their geometrical arrangement: chain-type, lattice-type, and hybrid-type. Chain-type and hybrid-type robots are particularly suitable for applications requiring coordinated locomotion [17]. Li [18] simplified the motion of caterpillars into a chain-type structure and analyzed the stability in relation to the formation and propagation of the “hump” shape. Chen [19,20] further divided caterpillar locomotion into three stages and formulated it as an end-effector tracking problem. While some segmented robots are designed as soft robots [21,22], which better replicate the flexibility of actual caterpillars [23], they tend to be slower and have increased control complexity. Most rigid-body robots mimicking caterpillar locomotion are currently chain-type, but due to the inability of single segments to compress or extend, they face limitations in motion performance [24]. Moreover, many have not yet undergone comprehensive environmental testing.

Parallel mechanisms are increasingly employed as modular subsystems in various robots due to their superior stiffness, high payload-to-weight ratio, and dynamic properties [25]. However, there have been no instances of such mechanisms successfully replicating caterpillar-like movement. Liu proposed a serial–parallel hybrid worm-like robot with nine degrees of freedom, constructed from two symmetrically arranged 3-RPS parallel mechanisms, providing a quantitative analysis and control framework for each gait [26]. Wei [27] introduced a novel snake robot design based on a 3-RSR parallel mechanism. Both designs, however, exhibit relatively slow speeds and require different quantitative analyses for each gait. Bi [11] proposed a 6-segment robot with a telescopic module and rotating joints in series and introduced a unified gain control framework to simplify the control logic by unifying the gait signals for worm-like and snake-like locomotion modes. Most existing research focuses on mechanical design and quantitative gait analysis or proposes unified gait generation methods at a signal level. However, there is still no comprehensive gait generation method that fully leverages the hyper-redundancy of these structures, nor a unified framework that can transform segmented robot locomotion into an end-effector tracking problem to enhance the motion performance of multi-segment robots.

For hyper-redundant continuum snake-like robots with fixed bases like [28,29,30], task space controllers (i.e., controlling the Cartesian position and orientation of the end-effector) act directly on the robot’s motion target, providing robustness against model uncertainties [31]. The navigation problem of continuous robots is simplified into a trajectory-tracking problem by discretizing a curve with a fixed step into an ordered point cloud [32,33]. However, mobile robots are necessary for specific exploration tasks covering longer distances. In the case of mobile multi-section robots, a similar solution approach can be achieved by determining which segments need to track specific trajectories to generate particular robot motion effects. Conversely, there are greater possibilities for various gaits on segmented robots with more degrees of freedom in serial–parallel hybrid configurations. A unified gait framework to quantitatively analyze their gaits and transform segmented robot locomotion into end-effector tracking problems can provide a convenient condition for optimizing specific gait motion performance and trajectory tracking problems in subsequent research [34,35].

This paper presents an SSU-based gait generation method to take advantage of the redundancy in the serial–parallel hybrid structure, aiming to enhance the robot’s performance. The contributions of this work are fourfold: (1) By abstracting segmented animals into a 6-DOF rigid body and employing the proposed stable segment update (SSU) concept, the SSU method is introduced to quantitatively analyze caterpillar locomotion. (2) The kinematic characteristics of a multi-segment 4-3-RSR robot are established and analyzed, formulating the motion problem as an end-effector tracking problem. (3) A method for selecting parameters for applying the SSU to multi-segment robots with fewer degrees of freedom is provided, including three sets of gains generated for trajectory planning of the swinging segment. (4) Experimental validation demonstrates that the SSU method can generate multiple gaits with varying speeds and adaptability across unstructured terrain.

## 2. Methods

### 2.1. Natural Caterpillar Locomotion Pattern

**Figure 1:**
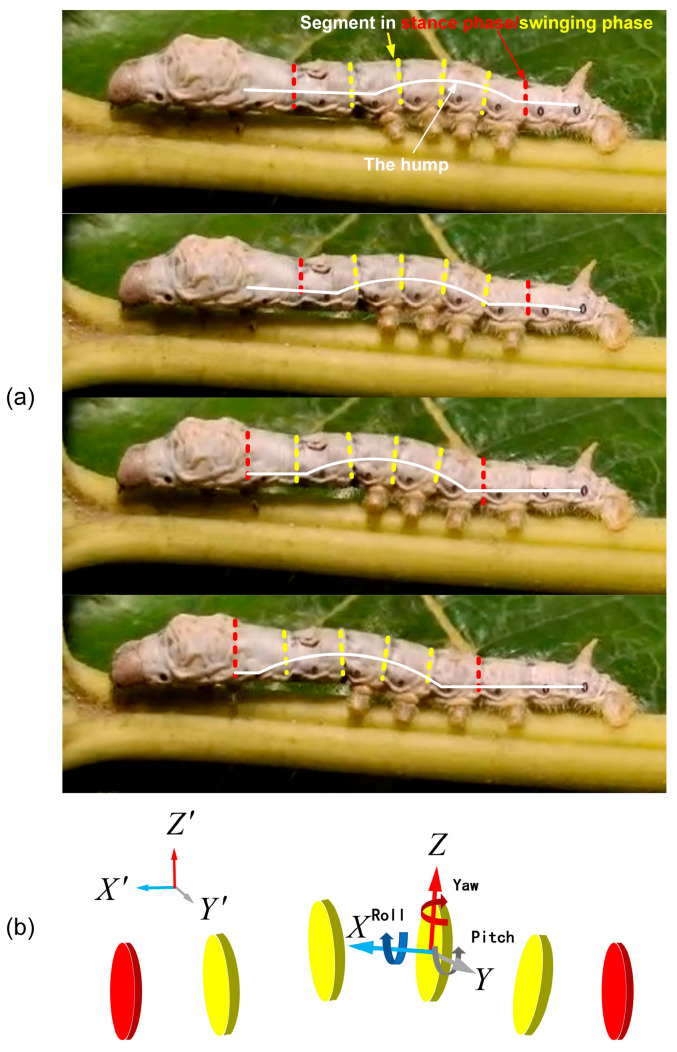
Natural caterpillar locomotion pattern. (**a**) Natural caterpillar locomotion sequence (the red dashed line represents stable segment; the yellow dashed line represents swinging segment). (**b**) Schematic diagram of natural caterpillar segments.

When the caterpillar crawls forward, caterpillars form a traveling wave along the body from the tail to the head, making the hump move and the body crawl forward [20]. For each segment, the swinging and stance phases alternate in the step mode. As shown in Figure 1b, the body hump and segment pattern of natural caterpillars can be observed by abstracting the single segment of the caterpillar as a rigid body with a 6 DOF. As shown in Figure 1a, we can observe the number and position of the segments in the swinging phase at an instant time when the hump is formed. Consequently, this periodic motion is divided into two parts.

**Figure 2:**
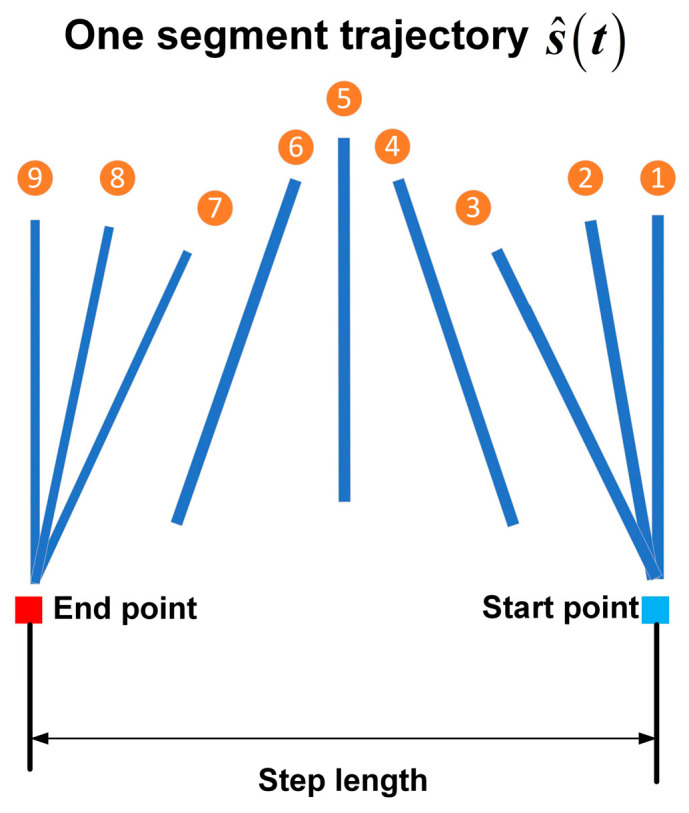
Nine-state of one segment motion trajectory.

The movement of silkworms belonging to the natural caterpillar species also includes these characteristics. Figure 1 shows a series of images illustrating the locomotion pattern witnessed on silkworms, and one part is a single-segment motion trajectory. The caterpillar reaches a state close to vertical with the ground at the midpoint of the movement cycle for each segment, with the other segments showing variations in pitch angle [13]. As shown in Figure 2, the trajectory can be simplified into three stages, representing the process of forward locomotion:

Preparatory lifting stage (1–3 state): The support foot contacts the substrate, and the segment pitch angle changes from zero to positive.

Lifting stage (4–6 state): The segment is lifted from the substrate to the highest point and moves forward, and the pitch angle changes from positive to zero. Then, the segment is lowered from the highest point to the substrate and moves forward, and the pitch angle changes from zero to negative.

Resetting stage (7–9 state): The support foot contacts the substrate, and the segment pitch angle changes from negative to zero.

In another part, as shown in Figure 1, the hump is caused by the same periodic motion trajectory, but the different phase φ in adjacent segments when the motion period is T. Figure 1 shows that the swinging and stance phases alternate in the step mode.

(Note that: At each moment, each segment is at a different position along the trajectory. The phase difference between adjacent segments in the same trajectory motion is expressed as φ. The time for tracking the single-segment trajectory is T.

### 2.2. SSU Gait Generation Method Based on Caterpillar-like Locomotion

The bio-inspired caterpillar-like gait generation method consists of a single-segment trajectory which needs single-segment to track and determine when to start tracking the trajectory. Similarly to the caterpillar, the multi-segmented robot enables us to simulate the redundancy property of natural caterpillars’ locomotion.

The hump moves forward as some segments of the stance phase become the swinging phase, and Figure 1 shows that the yellow segment is in the swinging phase, and the red segment is in the stance phase. At an instant time, a segment (from the swinging phase to the stance phase or the stance phase to the swinging phase) is about to undergo state transformation, which serves as the main entry point for us to analyze the motion mode of caterpillar-like locomotion.

**Figure 3:**
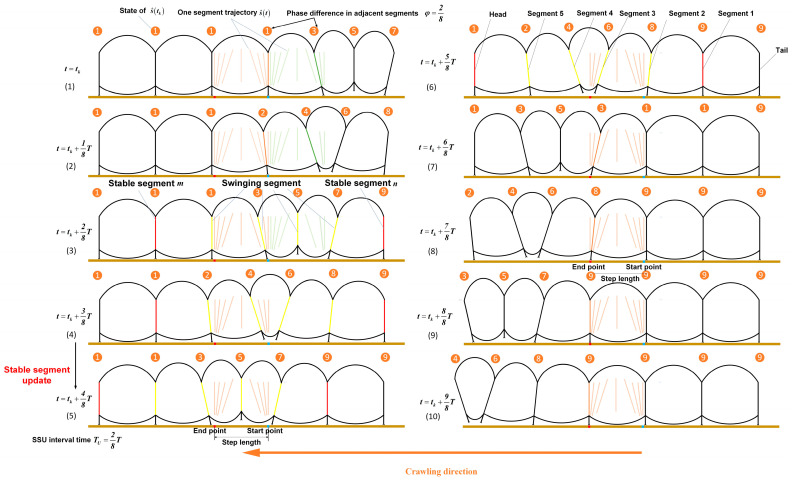
The hump formed on the natural caterpillar locomotion in a single segment 9-state trajectory. The illustration of the hump formed in the SSU method (red segment ((**3**)–(**6**) left) is the segment that is about to enter the swinging phase during the stance phase; red segment (right) is the segment that has ended the swinging phase during the stance phase. The yellow segment is the swinging segment in the swinging phase).

A caterpillar consists of $N_{\max}$ segments crawling at an instant time $t_{k}$. There are two of the nth segments and mth segments, where m>n, so the segments between the mth segment and nth segment are always in swinging phase. Other segments are stationary relative to the substrate. Then, at instant time $t_{k}$, the mth and nth segment are the stable segments (see Figure 3(3)–(6) red segment) and the $\left(n+1\right)\mathrm{th}$, $\left(n+2\right)\mathrm{th}$, …, $\left(m-2\right)\mathrm{th}$, $\left(m-1\right)\mathrm{th}$ segment as the swinging segment (see Figure 3(3)–(6) yellow segment) are designated.

In this paper, assume that all segments have the same trajectory period. For each caterpillar-like locomotion stride, the locomotion problem can be posed as an end-effector tracking problem. We then present the SSU gait generation method and end-effector tracking problem for realizing the caterpillar-like locomotion as follows:

(1) Initialize the stable segment, set m=0, $n=m-N_{S}-1$, and K=0, where m and n are the stable segments of the caterpillar, $N_{S}$ is the number of segments in the swinging phase of the gait, and K is a constant used to determine whether a gait has ended.
(2) At instant time $t_{k}=\left(T_{U}\right)TK$, the stable segments update once,
  (1) m=m+λ,n=n+λ
  where $T_{U}$ represents the time interval during which the caterpillar alternates between the stable phase and the swinging phase, and it is referred to as the stable segment update time. λ is number of finished progressive swinging segments for each SSU interval $T_{U}$ and $T_{U}$ can be given by
  (2) $$T_{U}=\left\{\begin{array}{c} T,if\varphi =1\\ \varphi T,if\varphi \in \left(0,1\right) \end{array}\right.$$
  When φ=1, the previous segment completes the entire trajectory before the next segment starts moving. $T_{U}$ is the time interval from when the previous segment starts track $\hat{s}\left(t\right)$ to when the next segment begins to track $\hat{s}\left(t\right)$.
(3) Swinging segment begins to track the trajectory $\hat{s}\left(t\right)$;
(4) One stride completion judgment:
  (a) K=K+1;
  (b) If n≠N, return to Step (2);
  (c) One stride ends, and return to Step (1).

**Figure 4:**
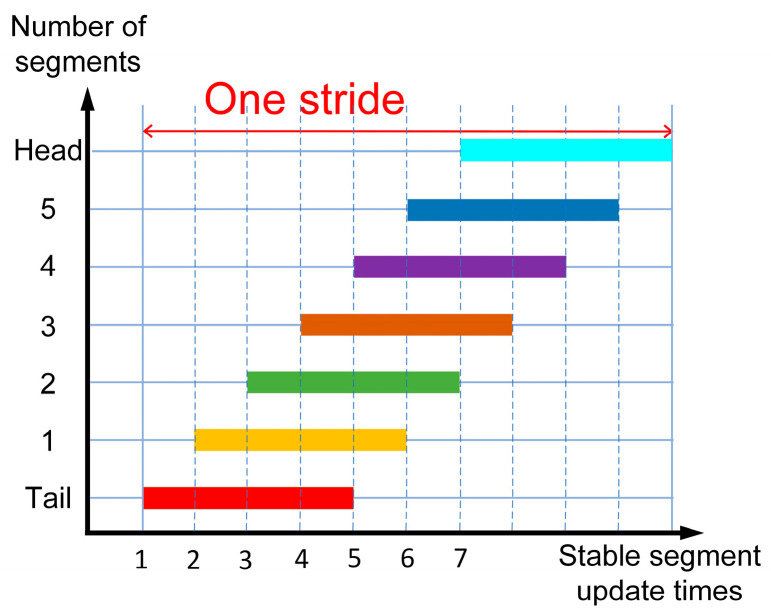
Footfall-pattern diagram of nature caterpillar gait.

The locomotion characteristics of caterpillars (see Figure 3) can be described using the SSU method, with the SSU parameters N=7, φ=1/4, $N_{S}=4$, $T_{U}=1/4T$, and λ=1. In Figure 3(1)–(10), the progression from the right (see Figure 3(1)) to the left (see Figure 3(10)) is shown as the caterpillar moves forward by one step length. The trajectories of all the segments are represented by $\hat{s}\left(t\right)$ as mentioned in Figure 2, and each swinging segment differs in its motion cycle from the adjacent segments by two states at all times, i.e., a phase difference of φ=1/4. Figure 3(4,5) show the process of stable segment updates at $t=t_{k}+(4/8)T$, during which segment 5 begins to follow the trajectory $\hat{s}\left(t\right)$. The footfall pattern of the segments is Tail (0)-1-2-3-4-5-Head (6) (see Figure 4).

For a segmented caterpillar robot, the fewer segments moved each time, the more stable the movement of the other segments connected to the ground, that is, $N_{S}=N_{\min}$. The time required for the robot to complete one gait is $N_{\max}T_{U}+T$ (update the time $N_{\max}T_{U}$ for the last segment, adding the time T it takes for that segment to complete its motion), and reducing the phase difference can decrease the update time, allowing the robot to progress more swing segments in the same amount of time. Additionally, we introduced the parameter λ in the SSU method to increase the adaptability of this movement pattern to the robot. In natural caterpillar movement, λ=1 implies that during a segment update, one segment completes tracking its trajectory. For a robot with insufficient degrees of freedom for a single segment, there may be no kinematic solution when $N_{S}=1$, similar to the analysis of the 4-3-RSR robot in Section 3.2 of this paper. To achieve the condition $N_{S}=N_{\min}$, introducing λ can simplify this problem, similar to determining various SSU method parameters based on the 4-3-RSR robot in Section 4 of this paper. By making appropriate selections based on the robot’s structure, the desired motion target can be achieved.

### 2.3. Robot Modeling

Here, we introduce the series-parallel hybrid segmented robot, the 4-3-RSR robot configuration, and its system kinematics modeling and analysis based on which the SSU method developed in Section 2 can be applied.

The main content of Section 2 involves abstracting the motion of a caterpillar into the motion relationships of several segments and using several parameters to describe this pattern. Similarly to the control problem of a serial–parallel hybrid segmented robot, with each segment being a parallel manipulator, the motion problem of a single segment can be transformed into an end-effector tracking problem of a single parallel manipulator, thereby achieving more precise control of the robot’s motion. Ignoring the requirement for speed, in terms of motion stability, the robot’s most stable gait is moving one segment at a time. In current work [19] simulating caterpillar motion, when a single segment has 2 DOF, $N_{S}=4$. On the other hand, according to [24], a caterpillar’s motion involves bending and extending to increase motion speed. Therefore, a 3-RSR parallel mechanism as the single segment of the segmented robot was chosen to test how the gait generated by applying a SSU to segmented robots performs in speed and stability.

#### 2.3.1. 4-3-RSR Forward Kinematics

**Figure 5:**
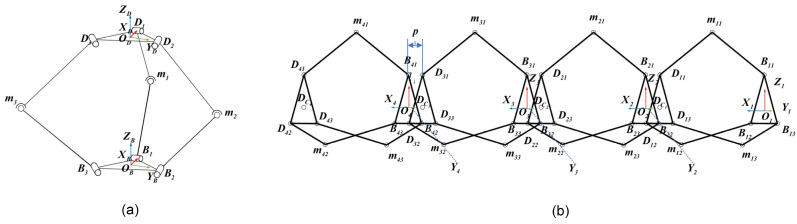
Robot mechanism and variables. (**a**) 3-RSR. (**b**) 4-3-RSR.

As shown in Figure 5, the caterpillar robot consists of four identical 3-RSR parallel structures connected in series. The kth coordinate system $O_{k}x_{k}y_{k}z_{k}$, k=1,2,3,4, is attached to the ith base plate center $B_{C_{i}}$, the ith distal half-joint, mid-joint, and base half-joint denoted as $D_{i,j},m_{i,j},B_{i,j}$, i=1,2,3,4, j=1,2,3.

The $D_{i,j}$ positions in $O_{k}x_{k}y_{k}z_{k}$ can be given by

(3) DCik=DCxikDCyikDCzikT

where superscript k denotes the kth coordinate system, the superscript *T* is the matrix transposition, and DCxik, DCyik, DCzik is the $D_{i,j}$ in XYZ axis component in $O_{k}x_{k}y_{k}z_{k}$.

The vector coordinate system $X_{i}Y_{i}Z_{i}$ axis in $O_{k}x_{k}y_{k}z_{k}$ can be given by

(4) Xi+1k=Di,1k−DCikDi,1k−DCik,Zi+1k=DnikDnik,Yi+1k=Xik×ZikXik×Zik

where $X_{1}=\left[\begin{array}{ccc} 1 & 0 & 0 \end{array}\right],Y_{1}=\left[\begin{array}{ccc} 0 & 1 & 0 \end{array}\right],Z_{1}=\left[\begin{array}{ccc} 0 & 0 & 1 \end{array}\right]$.

The ith coordinate system to the $\left(i+1\right)\mathrm{th}$ coordinate system homogeneous coordinate transformation matrix can be given by

(5) Ti+1i=Xi+1i⋅XiiYi+1i⋅XiiZi+1i⋅XiiDCxii+pDnixiDniiXi+1i⋅YiiYi+1i⋅YiiZi+1i⋅YiiDCyii+pDniyiDniiXi+1i⋅ZiiYi+1i⋅ZiiZi+1i⋅ZiiDCzii+pDniziDnii0001

where p is the distant of the $\left(i+1\right)\mathrm{th}$ base plate to the ith distal plate, and T10 is unit matrix.

The ith mid plate normal $N_{i}$, center of distal plate $D_{C_{i}}$, and normal to distal plate $D_{n_{i}}$ in $O_{k}x_{k}y_{k}z_{k}$ can be given by

(6) Nik=mi,2k−mi,1k×mi,3k−mi,1k

(7) DCik=Di,1k+Di,2k+Di,3k3

(8) Dnik=Di,2k−Di,1k×Di,3k−Di,1k

The $B_{i,j},m_{i,j},D_{i,j}$ in $O_{k}x_{k}y_{k}z_{k}$ can be given by

(9) Bi,jk=∏n=k+1iTnn−1B1,j1mi,jk=∏n=k+1iTnn−1m1,j1Di,jk=∏n=k+1iTnn−1D1,j1

When i=k, Bi,ji,mi,ji,Di,ji can be given by 3-RSR forward kinematics [36]:

(10) $$B_{1}=\left[\begin{array}{ccc} \frac{b}{\sqrt{3}} & 0 & 0 \end{array}\right],B_{2}=\left[\begin{array}{ccc} -\frac{b}{2\sqrt{3}} & \frac{b}{2} & 0 \end{array}\right],B_{3}=\left[\begin{array}{ccc} -\frac{b}{2\sqrt{3}} & -\frac{b}{2} & 0 \end{array}\right]$$

(11) $$\begin{array}{l} m_{1}=\left[\begin{array}{ccc} l\cos\left(\theta_{1}\right)+\frac{b}{\sqrt{3}} & 0 & l\sin(\theta_{1}) \end{array}\right]\\ m_{2}=\left[\begin{array}{ccc} -\frac{l}{2}\cos\left(\theta_{2}\right)+\frac{b}{2\sqrt{3}} & \frac{l\sqrt{3}}{2}\left(\theta_{2}\right)+\frac{b}{2} & l\sin(\theta_{2}) \end{array}\right]\\ m_{3}=\left[\begin{array}{ccc} -\frac{l}{2}\cos\left(\theta_{3}\right)+\frac{b}{2\sqrt{3}} & -\frac{l\sqrt{3}}{2}\left(\theta_{2}\right)+\frac{b}{2} & l\sin(\theta_{3}) \end{array}\right] \end{array}$$

(12) $$D_{j}=B_{j}-2\frac{N\cdot \left(B_{j}-m_{j}\right)}{{\left\|N\right\|}^{2}}N$$

where $\;N=\left(m_{2}-m_{1}\right)\times \left(m_{3}-m_{1}\right)$ is the mid plate normal.

#### 2.3.2. The Determination of $N_{\min}$ for 4-3-RSR Robot

**Figure 6:**
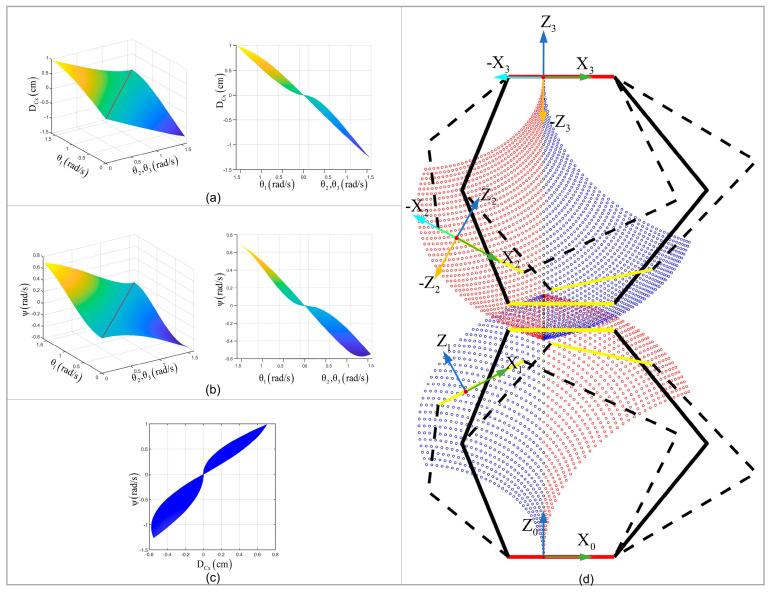
The kinematics analysis of 4-3-RSR robot SSU parameters $N_{\min}$. In the 3-RSR parallel mechanism, (**a**) the relationship of the distal plate center in axis X coordinate component and base angle $\theta_{1},\theta_{2},\theta_{3}$; (**b**) the relationship of the pitch angle of the distal plate and base angle $\theta_{1},\theta_{2},\theta_{3}$; (**c**) the relationship of the pitch angle of the distal plate and distal plate center in axis X coordinate component. (**d**) The 2-3-RSR mechanism and variables.

As shown in Figure 6a,b, for the 3-RSR structure under condition $\theta_{2}=\theta_{3}$, when $\theta_{1}>\theta_{2}$, $D_{Cx}>0,\psi >0$ can be obtained. When $\theta_{1}<\theta_{2},$ $D_{Cx}<0,\psi <0$ can be obtained (where $\psi =\arctan\left(\left(-X_{D}\cdot Z_{B}\right)/\left(Z_{D}\cdot Z_{B}\right)\right)$). Figure 6c shows $D_{Cx}$ and ψ meet the same positive and negative conditions X.

As shown in Figure 6d, ψ20>0 for the 3-RSR structure; the red part above meets DCx−2−3>0, ψ−2−3>0, and the blue part above meets DCx−2−3<0, ψ−2−3<0 (ψij and DCxij are the pitch angle and distal plate center axis component of $O_{i}x_{i}y_{i}z_{i}$ in $O_{j}x_{j}y_{j}z_{j}$).

For the 3-RSR structure, the blue part below meets DCx10<0, ψ10<0, the red part below is DCx10>0, ψ10>0, and the blue part above is ψ20<0 the red part above is ψ20>0.

Coordinate systems $O_{1}x_{1}y_{1}z_{1}$ and $O_{2}x_{2}y_{2}z_{2}$ have the same pitch angle due to their rigid connection within the shared workspace of the upper and lower 3-RSR structures (see the intersection of red and blue parts in Figure 6). Therefore, condition ψ10=ψ20 is a necessary and insufficient condition for a single segment to move. From the above analysis, it can be concluded that under m−n=2 condition, the $\left(m-1\right)\mathrm{th}$ segment can be moved when ψ10=ψ20=0.

#### 2.3.3. 2-3-RSR Inverse Kinematics

The two segments inverse kinematics solution needs to solve the relative position between the $O_{i}x_{i}y_{i}z_{i}$ and the $O_{i+2}x_{i+2}y_{i+2}z_{i+2}$ under the known relative position of the ith and the $\left(i+2\right)\mathrm{th}$ segment and solve all the base angles $\theta_{i,j},\theta_{i+1,j}$ of the ith and the $\left(i+2\right)\mathrm{th}$ segment.

The forward kinematics solution of the 2-3-RSR parallel structure can be given by

(13) DCi+2i=Tri+2iDCi+1i+2

because the origin of the $O_{i+2}x_{i+2}y_{i+2}z_{i+2}$ is $D_{C_{i+1}}$, i.e., DCi+1i+2=0001T and

(14) Tri+2i=r11rr12rr13rpxrr21rr22rr23rpyrr31rr32rr33rpzr0001

where Tri+2i is the known transformation matrix from the $O_{i}x_{i}y_{i}z_{i}$ to $O_{i+2}x_{i+2}y_{i+2}z_{i+2}$.

The center position of the $\left(i+1\right)\mathrm{th}$ distal plate $D_{C_{i+1}}$ in $O_{i}x_{i}y_{i}z_{i}$ can be given by

(15) DCi+1i=pxrpyrpzr

Let the unknown quantity DCii=DCxiiDCyiiDCzii be substituted into the 3-RSR inverse kinematics solution equation to obtain Di,jiDCii, DniiDCii, and DCii can be obtained from the 4-3-RSR forward kinematics:

The $O_{i+1}x_{i+1}y_{i+1}z_{i+1}$ can be represented by the unknown quantity DCii coordinate axis vector Xi+1i(DCii),Yi+1i(DCii),Zi+1i(DCii) in $O_{i}x_{i}y_{i}z_{i}$, and Ti+1iDCii.

Then, the center position of the $\left(i+1\right)\mathrm{th}$ distal plate $D_{C_{i+1}}$ in $O_{i}x_{i}y_{i}z_{i}$ can be given by

(16) DCi+1i+1DCii=DCi+1i−DCii⋅Xi+1iDCi+1i−DCii⋅Yi+1iDCi+1i−DCii⋅Zi+1i

According to DCi+1i+1=DCi+1xi+1DCi+1yi+1DCi+1zi+1, Di+1,ji+1DCii,Dni+1i+1DCii is obtained by substituting the 3-RSR inverse kinematics.

The Xi+2i+1(DCii), Yi+2i+1(DCii), Zi+2i+1(DCii) and Ti+2i+1DCii can also be expressed according to the 4-3-RSR forward kinematics.

The homogeneous transformation matrix from $O_{i}x_{i}y_{i}z_{i}$ to $O_{i+2}x_{i+2}y_{i+2}z_{i+2}$ can be given by

(17) Ti+2iDCii=Ti+1iDCiiTi+2i+1DCii

The unknown quantity DCii can be given by solving the equation:

(18) Tri+2i−Ti+2i=0

The base angle $\theta_{i,j},\theta_{i+1,j}$ can be given by substitute DCii,DCi+1i+1 into 3-RSR inverse kinematics [36].

### 2.4. Swinging Segment Trajectory

#### 2.4.1. The Determination of Gait Parameters for the 4-3-RSR Robot

From the above kinematics analysis, the 4-3-RSR cannot lift the $\left(i+1\right)\mathrm{th}$ segment under the condition that the ith and $\left(i+2\right)\mathrm{th}$ segments are stationary relative to the substrate. Our goals for designing gait parameters for the SSU and swinging segment trajectory are as follows:

(1) Improving robot stability by reducing the number of swinging segments.
(2) Avoiding workspace with unsolvable 3-RSR kinematics.
(3) Reducing ground friction to keep each step away from the ground.

**Figure 7:**
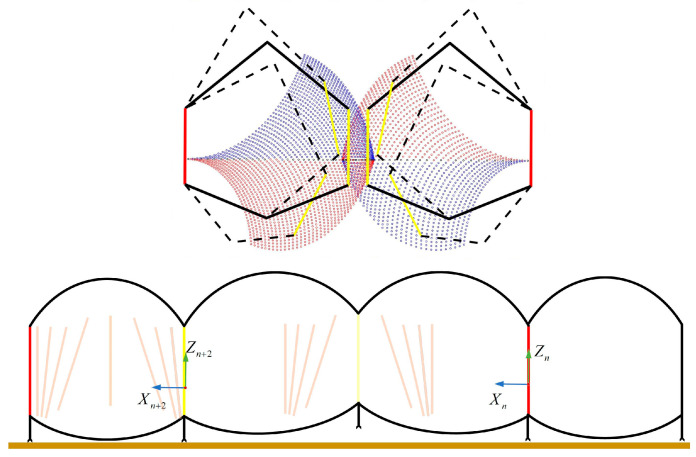
The robot posture when φ=1/2.

**Figure 8:**
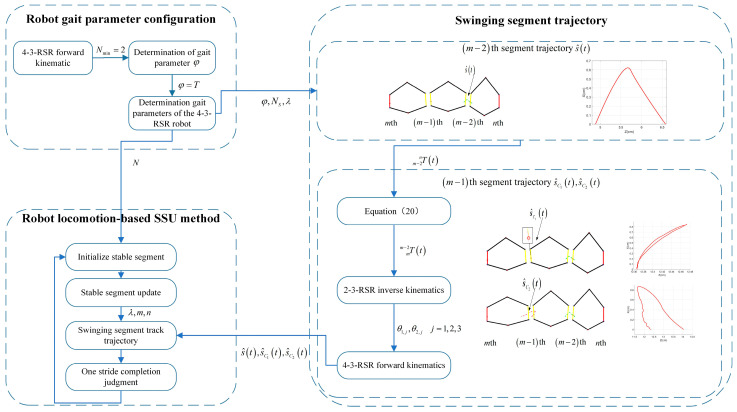
SSU gait generation flowchart.

According to the analysis in Section 2, $N_{S}$ can be given by

(19) $$N_{S}=\left\{\begin{array}{c} \frac{1}{\varphi },\varphi \in (0,1)\\ N^{*},\varphi =1 \end{array}\right.$$

where $N^{*}$ is the positive integer. As shown in Figure 7, when φ=1/2, but when $t_{k}=1/2T_{U}K$, there is no kinematic solution for this situation. So, we can make φ=1 and convert the continuous progressive humps into a discrete humps update at $t_{k}=T_{U}K$ to ensure the gait parameter goals. The step for generating gait is shown in Figure 8.

#### 2.4.2. The Determination of $\left(m-2\right)\mathrm{th}$ Segment Trajectory

In the height of each step $H_{step}$, length of each step $L_{step}$, and segment contraction length $C_{L}$, the motion trajectory $\hat{s}\left(t\right)$ can be calculated as Table 1 and Equation (20):

(20) $$\begin{array}{l} x\left(t\right)=a_{0}+a_{1}t+a_{2}t^{2}+a_{3}t^{3}\\ z\left(t\right)=b_{0}+b_{1}t+b_{2}t^{2}+b_{3}t^{3} \end{array}$$

#### 2.4.3. The Determination of $\left(m-1\right)\mathrm{th}$ Segment Trajectory

**Figure 9:**
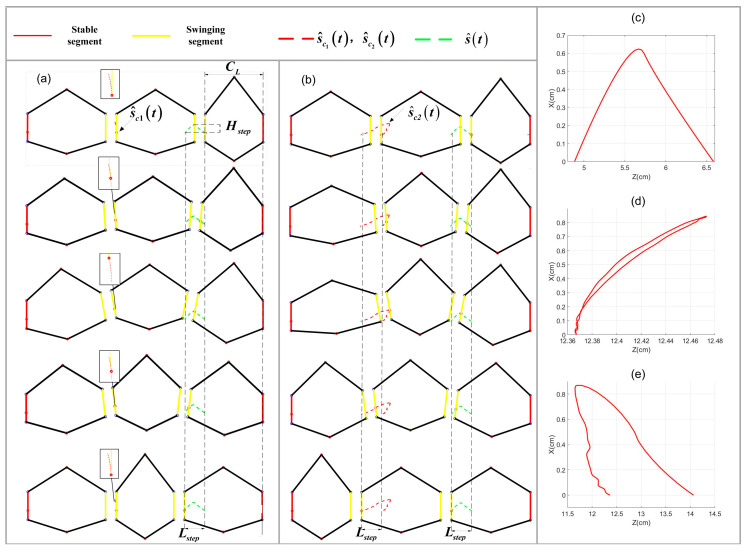
The SSU swinging segment trajectory. (**a**) The gaits sequence for $\left(m-1\right)\mathrm{th}$ segment trajectory ${\hat{s}}_{c_{1}}\left(t\right)$. (**b**) The gaits sequence for $\left(m-1\right)\mathrm{th}$ segment trajectory ${\hat{s}}_{c2}\left(t\right)$. (**c**) The trajectory of $\left(m-2\right)\mathrm{th}$ segment progressive. (**d**) The compensate trajectory ${\hat{s}}_{c1}\left(t\right)$ of $\left(m-1\right)\mathrm{th}$ segment. (**e**) The compensate trajectory ${\hat{s}}_{c2}\left(t\right)$ of $\left(m-1\right)\mathrm{th}$ segment progressive.

At an instant time $t_{k}$, as shown in Figure 9c, the position of the trajectory at $\hat{s}\left(t_{k}\right)$ and the position of the compensate trajectory at ${\hat{s}}_{c}\left(t_{k}\right)$ can be calculated as follows:

In order to make the stable segments m,n stationary relative to the substrate, the homogeneous transformation matrix of coordinate system $O_{n+1}x_{n+1}y_{n+1}z_{n+1}$, $O_{m}x_{m}y_{m}z_{m}$ attached on the swinging segment $\left(n+1\right)\mathrm{th},m\mathrm{th}$ at instant time $t_{k}$ are given by

(21) Tmn+1=T−1n+1nTrmn

where Tn+1n can be calculated by $\hat{s}\left(t_{k}\right)$, and Trmn can be calculated by the relative position of the stable segments m,n.

Substituting Tmn+1 into 2-3-RSR inverse kinematics yields base angle $\theta_{m-1,j},\theta_{m,j}$. Substituting $\theta_{m-1,j},\theta_{m,j}$ into 4-3-RSR forward kinematics yields s^ctk=DCn+1n.

### 2.5. Multi-Segment Robot Locomotion-Based SSU Method

As Equation (17) has multiple solutions, different search intervals can be selected to generate different compensation trajectories to complete the progression of one or two swinging segments in $T_{U}$ when solving the numerical solution. The progress of one swinging segment $\left(m-1\right)\mathrm{th}$ trajectory is denoted as ${\hat{s}}_{c1}\left(t\right)$ (see Figure 9d), and the progress of the second swinging segment $\left(m-1\right)\mathrm{th}$ trajectory is denoted as ${\hat{s}}_{c2}\left(t\right)$ (see Figure 9e).

Then, we can design the SSU method for the 4-3-RSR robot. The $\left(m-2\right)\mathrm{th}$ segment motion trajectory is $\hat{s}\left(t\right)$, the $\left(m-1\right)\mathrm{th}$ segment motion trajectory is ${\hat{s}}_{c1}\left(t\right)$ or ${\hat{s}}_{c2}\left(t\right)$, and the trajectory period is T; the SSU parameters are N=5, $N_{S}=2$, φ=1, $T_{U}=T$.

When the last update time $\left(m-1\right)\mathrm{th}$ segment track trajectory is ${\hat{s}}_{c1}\left(t\right)$, λ=1 can be determined. When the last update time $\left(m-1\right)\mathrm{th}$ segment track trajectory is ${\hat{s}}_{c2}\left(t\right)$, λ=2 can be determined.

**Figure 10:**
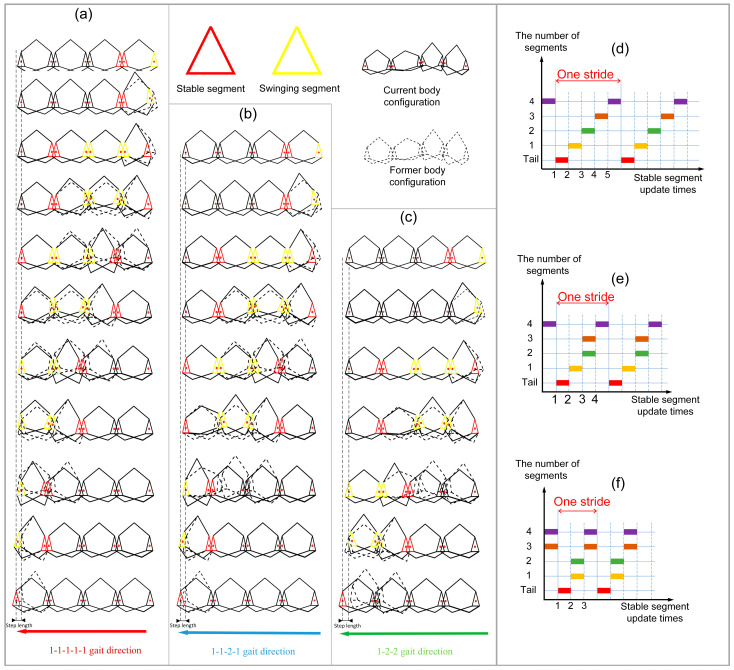
Three gaits pattern of 4-3-RSR robot. (**a**) The 1-1-1-1-1 gait, (**b**) 1-1-2-1 gait, and (**c**) 1-2-2 gait. Footfall-pattern diagram of the (**d**) 1-1-1-1-1 gait, (**e**) 1-1-2-1 gait, and (**f**) 1-2-2 gait.

After each update of the stable segment, the $\left(m-1\right)\mathrm{th}$ segments can choose to progress or be left in situ (see Figure 9a,b), and the 4-3-RSR robot with five segments can generate three gaits:

(1) 1-1-1-1-1 gait crawling locomotion: The $\left(m-1\right)\mathrm{th}$ segment selects compensated trajectory ${\hat{s}}_{c1}\left(t\right)$. The stable segment needs to be updated 5 times, and all segments take 5T to finish progress (see Figure 10a), with the footfall pattern of segment tail-1-2-3-4(head) (see Figure 10b).
(2) 1-1-2-1 gait crawling locomotion: When K=2, the $\left(m-1\right)\mathrm{th}$ segment selects segment compensated trajectory ${\hat{s}}_{c2}\left(t\right)$. The stable segment needs to be updated 4 times, and all segments take 4T to finish progress (see Figure 10b), with the footfall pattern of segment tail-1-2,3-4(head) (see Figure 10e).
(3) 1-2-2 gait crawling locomotion: When K≠0, $\left(m-1\right)\mathrm{th}$ segment selects compensated trajectory ${\hat{s}}_{c2}\left(t\right)$. The stable segment needs to be updated 3 times (see Figure 10c), and all segments take 3T to finish progress, with the footfall pattern of segment tail-1,2-3,4(head) (see Figure 10f).

In addition, when K=0, $\left(m-1\right)\mathrm{th}$ chooses tracking $\hat{s}\left(t\right)$ instead of track ${\hat{s}}_{c2}\left(t\right)$, which can improve the stability of tail motion.

## 3. Results

In this section, experiments on 4-3-RSR robots are performed to demonstrate the proposed SSU method for multi-segment robot locomotion.

**Figure 11:**
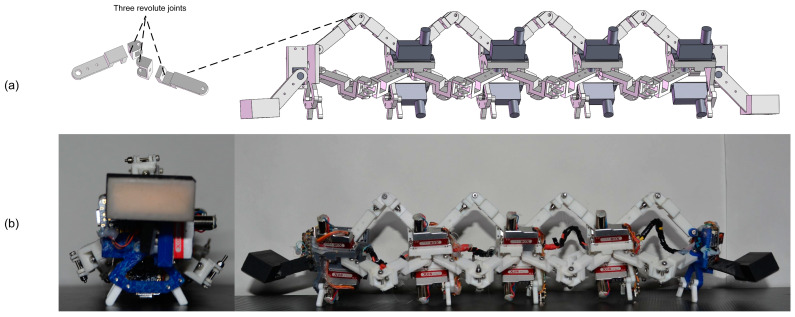
The 4-3-RSR robot. (**a**) Three rotary joints replace the sphere joint. (**b**) The 4-3-RSR robot press plate (left) and main view (right).

The entire body of the robot is made of 3D printing. As mentioned in Section 3 and Figure 11a, the 3D printing 4-3-RSR robot with spherical joints comprises three rotary joints instead. Table 2 lists the main physical parameters of the robot, in which the length of the robot when all segments are fully extended is 42 cm, and the length of each segment when fully retracted is also 30 cm. To simulate the dynamic anchoring effect of the head and tail of a natural caterpillar during movement, we designed two plates with silicone coatings on their bottom surfaces to increase friction and provide dynamic anchoring. Each plate was driven by a servo motor (see Figure 11b). A small chip, Seeed Studio XIAO SAMD21 (Seeed Technology, Shenzhen, China), was installed in each segment to send PWM signals to drive the three servo motors of that segment. The same chip was also placed in the head, functioning as the host to communicate with each segment via IIC bus, sending the required motion angles for that moment. The entire control process was an open-loop control system, where joint trajectories calculated from inverse kinematics were transmitted to the motor servo controllers.

Table 3 lists the crawling pattern parameters of the three gaits calculated in Section 4. The generated trajectories of the ${\hat{s}}_{c1}\left(t\right)$ and ${\hat{s}}_{c2}\left(t\right)$ are solved by Particle Swarm Optimization (PSO) for Equation (14), where $u_{b}$ is the PSO constraint upper bound, $u_{b}$ is the PSO constraint lower bound, the $X_{D_{C}}$ is the initial value of the *X*-axis search, and λ is the range of search. By selecting different $X_{D_{C}}$ sequences according to λ to make the $\left(m-1\right)\mathrm{th}$ segment progressive or non-progressive, the empirical range of the *Z*-axis search is set to [−1.5, 1] to reduce PSO computation time, substituting the expected coordinate range into Equation (13) for solving

(22) minimizeθm−1,j,θm−2,j Trmn+1−Tmn+1

The base angle can be obtained, and by substituting it into the 4-3-RSR forward kinematic solution, ${\hat{s}}_{c1}(t)$ and ${\hat{s}}_{c2}(t)$ can be obtained.

**Figure 12:**
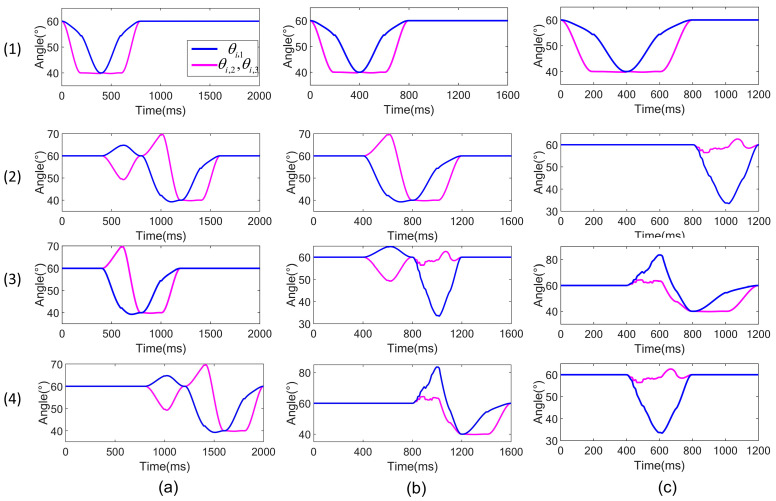
Joint trajectories of 4-3-RSR robot. (**a**) The 1-1-1-1-1 gait, (**b**) 1-1-2-1 gait, and (**c**) 1-2-2 gait, where (1) (2) (3) (4) illuminate the 1st, 2nd, 3rd, and 4th 3-RSR parallel mechanism joint trajectories.

In crawling locomotion numerical simulations, the trajectories of the joints of three gait locomotion are illustrated in Figure 12.

### 3.1. Motion Performance

The robot’s motion is tested according to the gait (see Figure 10).

#### 3.1.1. Three Caterpillar-like Locomotion Gaits

**Figure 13:**
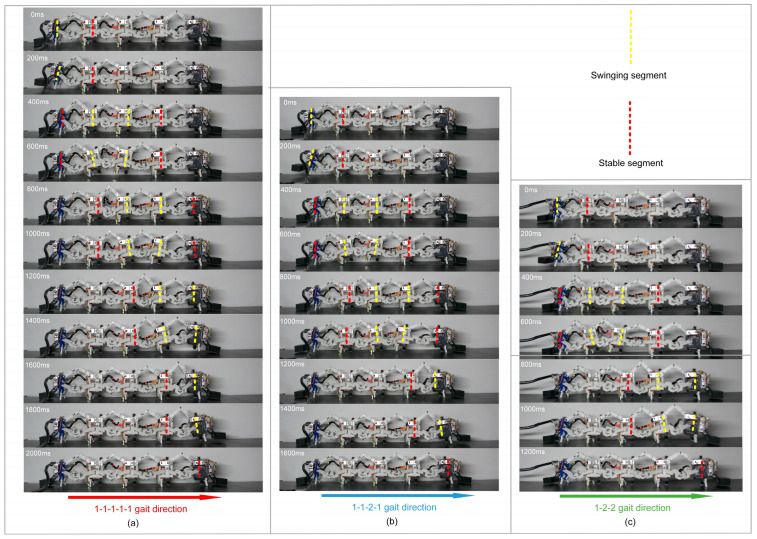
Three gaits experiment of the 4-3-RSR robot. (**a**) The 1-1-1-1-1 gait, (**b**) 1-1-2-1 gait, and (**c**) 1-2-2 gait. (The red dotted line represents the stable segment, and the yellow dotted line represents the swinging segment).

As shown in Figure 13, due to the lack of a dynamic model and a substrate friction model, the reaction force when each segment moves forward causes the entire robot to rebound and move backward, which is most evident in the segment tracking ${\hat{s}}_{c2}(t)$ of 1-1-2-1 and 1-2-2 gaits when T=40 ms. Table 4 shows the experiment speeds for the three gaits at T=40 ms were 22.93 $\mathrm{mm}\cdot s^{-1}$ (1-1-1-1-1 gait), 22.74 $\mathrm{mm}\cdot s^{-1}$ (1-1-2-1 gait), and 22.62 $\mathrm{mm}\cdot s^{-1}$ (1-2-2 gait), and the reaction force of trajectory ${\hat{s}}_{c2}(t)$ was more extensive than T=400 ms, leading the robot to rebound larger, and the approximation of three gait speeds. However, when T=400 ms, the 1-2-2 gait and 1-1-2-1 gait were 45.17% and 17.76% faster, respectively, compared with the 1-1-1-1-1 gait.

Table 5 gives the result of caterpillar-inspired robots in other relevant works where the *NV* (normalized velocity) is derived to compare the robots with different mechanical mechanisms. NV is given by [37] $NV=velocity/bodylength$. As shown in Table 5, the NV of the discrete traveling wave gait generated by the SSU in our proposed method on the 4-3-RSR robot is superior to that achieved in most other studies. Currently, the parameters for the single-segment trajectory are obtained from experimental tests, and numerical solutions within the robot’s workspace are determined using PSO.

#### 3.1.2. Rectilinear-Like Gait

**Figure 14:**
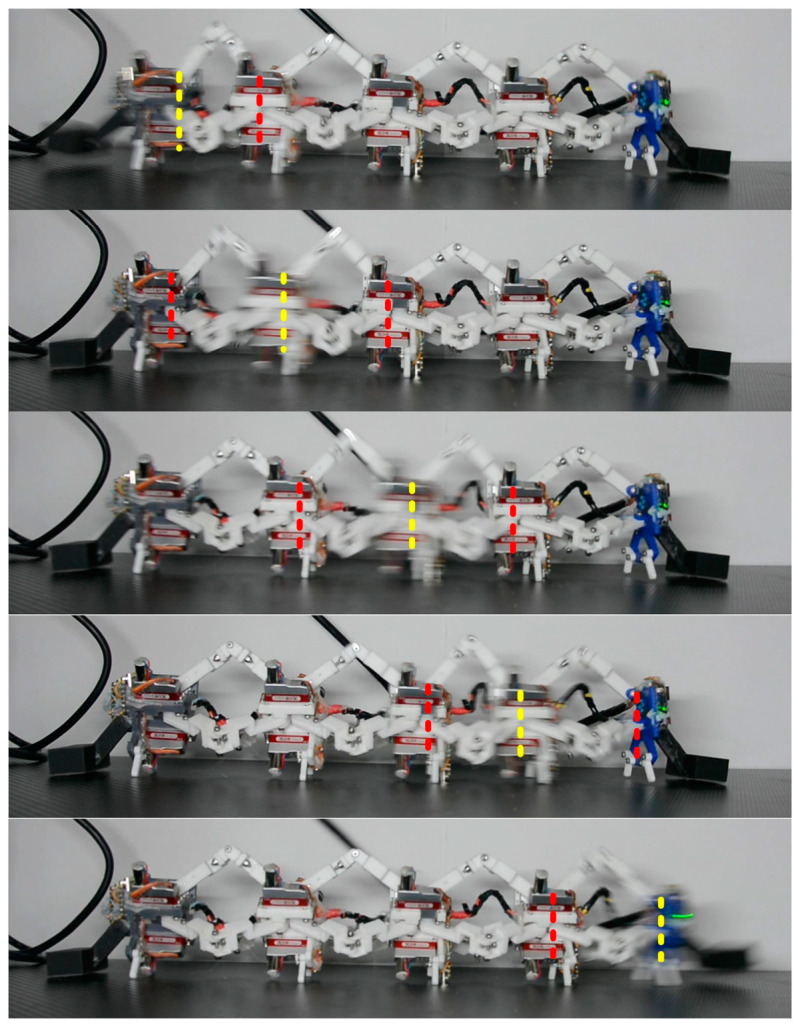
Locomotion of the 4-3-RSR robot rectilinear gait.

As analyzed in Section 4, only when ψ10=ψ20 can a segment be moved, selecting the gait planning parameters ($H_{step}=0,L_{step}=18.7,C_{L}=43.6$) by experiments. Under these circumstances, the head and tail pressing plates are alternatively pressed down to imitate earthworm locomotion in which the setae extend so that the segment can anchor against the surface with high friction [39]. The head’s pressing plate lifts up when n=4 and the tail’s pressing plate lifts up when m=0. The trajectory period is T, and the SSU parameters are N=5, $N_{S}=1$, λ=1, and $T_{U}=T$. As shown in Figure 14, the speed that can be achieved in rectilinear-like gait was 43.43 $\mathrm{mm}\cdot s^{-1}$.

Comparison of the locomotion speed of the rectilinear gaits experiment on flat ground in our research with other relevant works is presented in Table 5. As shown in Table 5, the *NV* under the experimental parameters was superior to or comparable to the relevant studies.

### 3.2. Environmental Adaptability

#### 3.2.1. Crossing Gaps

Caterpillars should be able to crawl through gaps to obtain the necessary food for survival. To compare the gait effects of the 4-3-RSR robot, we designed a gap with a width of 30 mm.

**Figure 15:**
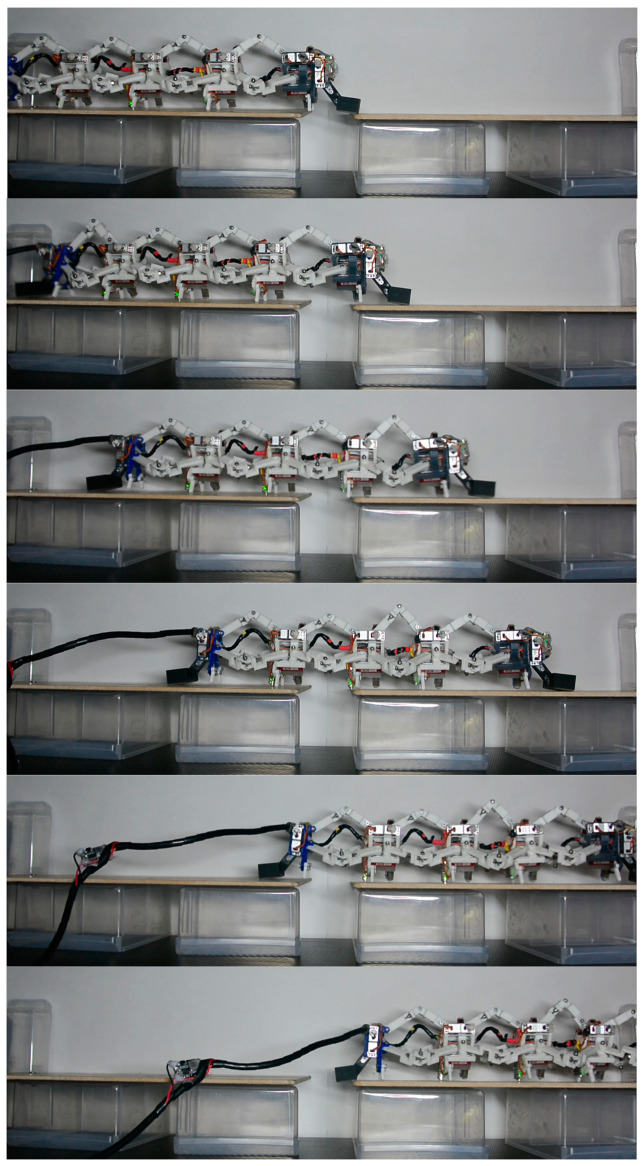
The 1-1-1-1-1-1 gait crossing gaps.

As shown in Figure 15, at the same angle as pressing down on the pressure plate, the 1-1-1-1-1 gait in T=400 ms successfully compensated for some of the drop caused by mechanical errors with the leg lift. The 1-1-2-1 and 1-2-2 gaits could not cross the gap due to insufficient lifting height of the second and first segments, respectively, because of the second segment of the 1-1-2-1 gait and the first segment of the 1-2-2 gait track trajectory ${\hat{s}}_{c2}(t)$ with a height of 3.53 mm at half of $L_{step}$, which was 56.03% of the ${\hat{s}}_{c1}(t)$ trajectory with a height of 6.30 mm at half of $L_{step}$. The decrease in height made it difficult to overcome the problem of large mechanical gap in the 3D-printed parts.

The robot in [26] had a length of 1800 mm and successfully crossed a gap with a width of 300 mm using a specific gap traversal strategy. We compared the crossing ability of our robot with this robot’s using *NCGW* (normalized crossing gap width), where NCGW=gap width/body length. The *NCGW* for the 1-1-1-1-1 gait was 0.1 and [26] was 0.16. Although this capability in this paper is lower, in this experiment, the robot walked blindly without gap perception, demonstrating higher robustness.

#### 3.2.2. Crawling on Rough Terrain

The crawling substrate of caterpillars is not always flat. In order to evaluate the robot’s gait over rough terrain, the robot was used to move forward on bentonite particles.

**Figure 16:**
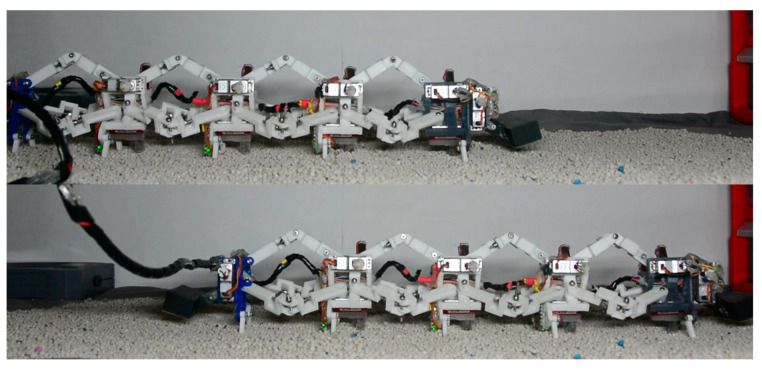
The 1-1-1-1-1-1 gait on roughness terrain.

As shown in Figure 16, with all the pressure plates raised and gait parameter T=80 ms, the experiment speed of the 1-1-1-1-1 type crawling gait was 6.14 $\mathrm{mm}\cdot s^{-1}$. The experiment speed of the 1-1-2-1 type crawling gait was 2.37 $\mathrm{mm}\cdot s^{-1}$. However, the 1-2-2 gait could not move forward on the bentonite particles, causing the entire robot to experience significant backward movement when tracking trajectory of segment 4.

Hence, although trajectory ${\hat{s}}_{c2}(t)$ brings speed improvement, the robot loses some stability due to backward swing. The NV of the serpentine gait in [40,41] in the rough terrain tests were 0.011 $\mathrm{body}\;\mathrm{length}\cdot s^{-1}$ and 0.03 $\mathrm{body}\;\mathrm{length}\cdot s^{-1}$, respectively. The NV of the 1-1-1-1-1 gait was 0.02 $\mathrm{body}\;\mathrm{length}\cdot s^{-1}$, higher than or comparable to relevant studies.

The unstructured terrain and max speed experimental results confirmed the effectiveness and stability of the proposed gait generation method for a multi-segments robot.

## 4. Discussion

Compared to traditional rigid-body robots inspired by caterpillar motion that is generated in single-segment non-expandable bodies, this paper applies the motion of a single-segment expandable caterpillar to robots, achieving speeds comparable to or better than those of caterpillar-inspired robots. Furthermore, the rigid-body caterpillar robot’s gait was adaptable in unexplored environments, such as crossing gaps and navigating rough, unstructured terrain.

In contrast to current multi-gait parallel–serial robot gait generation methods, this paper introduces a unified gait generation approach. Unlike the [26], where each gait has its unique formula, the SSU method generates each gait with different parameter sets. Compared with [11], this paper elevates gait generation from the signal level to the trajectory tracking problem of gaits composed of different parameter sets. On one hand, this enhances the optimization potential of gaits. For example, the gaits generated in this paper differ from other caterpillar-inspired and natural caterpillar gaits, as their peaks are discrete. On the other hand, it simplifies the motion planning required for new robot movements. It is worth mentioning that we have already validated SSU on a single-segment six-degree-of-freedom Stewart platform and achieved superior motion performance.

Although the proposed SSU model effectively translates caterpillar-like motion into an end-effector tracking problem and maps it onto a robot, generating various gaits based on different SSU parameters, the gaits produced by the SSU method are limited to discrete step-by-step movements. They cannot generate continuous motions similar to the serpentine gait, resulting in significantly lower efficiency in terrain-agnostic locomotion compared with the serpentine gait. On the other hand, the more degrees of freedom a single segment has, the wider the workspace for planning SSU parameters and single-segment trajectories. In the analysis of the minimal swing segment $N_{S}$ conducted in Section 3.2 of this paper, it is evident that in the serial–parallel hybrid robot composed of a Stewart platform with a single segment of six degrees of freedom, $N_{S}=1$. In contrast, segmented robots with fewer degrees of freedom segments will face a significantly greater challenge in finding viable solutions for SSU parameters and single-segment trajectories than robots with higher degrees of freedom in each segment.

The 1-1-1-1-1 gait presented in this article has limited blind walking adaptability and significantly reduced speed. For more complex environments, it is worth considering optimizing the trajectory of the single segment under a specific SSU gait parameter to adapt to the surface curvature of different terrains. On the other hand, in high-speed locomotion, the speed significantly decays due to the neglect of friction between the robot’s foot and the ground. In the future, we plan to improve locomotion efficiency by considering the impact of friction when optimizing the trajectory of the single segment.

The research application concept and the main gait planning objectives in this paper are consistent with the advantages of caterpillars in nature. Due to the attachment of more segments to the ground during each movement, this type of motion is suitable for narrow terrains and especially well suited for climbing robots. Additionally, we plan to attach an adhesion device to the end of the robot segments to assess their climbing abilities, which have potential applications in earthquake relief and urban search and rescue missions.

## 5. Conclusions

This paper proposed a gait generation method SSU inspired by caterpillars for series-parallel hybrid segmented robots. By analyzing the movement patterns of caterpillars, the caterpillar structure is simplified to a rigid body with a 6 DOF, and the trajectory of individual segment movements and the motion relationships between segments are explored. This movement is transformed into an SSU composed of several vital parameters. The 4-3-RSR robot inverse kinematics solution of the 2-3-RSR and the forward kinematics solution of the 4-3-RSR are established to verify the effectiveness of the SSU method. According to the SSU method, three gait patterns are generated on the 4-3-RSR robot. The experiment verified that these three gait patterns have speed and can cope with unstructured terrain and proved that the SSU could generate multiple gait patterns for multiple segments to cope with various terrain patterns.

## Figures and Tables

**Table 1:** Parameters of $\left(m-2\right)\mathrm{th}$ segment trajectory.

Time (s)	x	y	z
$t_{0}=0$	0	0	$C_{L}$
$t_{h}=T/2$	$H_{step}$	0	0
$t_{f}=T$	0	0	$C_{L}+L_{step}$

**Table 2:** Parameters of 4-3-RSR.

Items	Value
Weight	202 g
k	9.0 mm
l	38.0 mm
b	25.1 mm
Length of Robot (Extend State)	420 mm
Length of Robot (Shorten State)	300 mm

**Table 3:** Parameters for gait planning.

Items	Value
$H_{step}$	6.50 mm
$L_{step}$	16.8 mm
$C_{L}$	48.8 mm
$u_{b}$	$\left[\begin{array}{ccc} 1 & 0 & Z_{D_{c}}+\varepsilon \end{array}\right]$
$l_{b}$	$\left[\begin{array}{ccc} -1.5 & 0 & Z_{D_{c}}-\varepsilon \end{array}\right]$
ε	0.001

**Table 4:** Velocity and normalized velocity.

Gait	*T* = 400 ms Velocity (mm/s)	*T* = 40 ms Velocity (mm/s)	Normalized Velocity (Body Length/s)	Normalized Crossing Gap Width (Gap Width/Body Length)	Normalized Velocity on Rough Terrain (Body Length/s)
1-1-1-1-1	4.54	22.93	0.0764	0.1	0.02
1-1-2-1	5.52	22.74	0.0758	×	0.008
1-2-2	8.28	22.62	0.0754	×	×
Rectilinear	×	43.43	0.145	×	×

**Table 5:** Comparison of normalized velocity.

Ref	Gait	Normalized Velocity (Body Length/s)	Normalized Crossing Gap Width (Gap Width/Body Length)	Normalized Velocity on Rough Terrain (Body Length/s)
[18]	Caterpillar	0.023	×	×
[17]	Caterpillar	0.045	×	×
[21]	Caterpillar	0.048	×	×
[38]	Caterpillar	0.01	×	×
[10]	Rectilinear	0.166	×	×
[27]	Rectilinear	0.03	×	×
[11]	Rectilinear	0.02	×	×
[39]	Rectilinear	0.129	×	×
[26]	Worm-like	0.016	0.167	×
[40]	Snake-like	×	×	0.011
[41]	Snake-like	0.2	×	0.034

## Data Availability

The raw data supporting the conclusions of this article will be made available by the authors on request.
